# Effects of Different Stimulation Conditions on the Stimulation Effect of Noisy Galvanic Vestibular Stimulation

**DOI:** 10.3389/fnhum.2020.581405

**Published:** 2020-09-24

**Authors:** Yasuto Inukai, Shota Miyaguchi, Miki Saito, Naofumi Otsuru, Hideaki Onishi

**Affiliations:** ^1^Department of Physical Therapy, Niigata University of Health and Welfare, Niigata, Japan; ^2^Institute for Human Movement and Medical Sciences, Niigata University of Health and Welfare, Niigata, Japan; ^3^Department of Rehabilitation, Kaetsu Hospital, Niigata, Japan

**Keywords:** vestibular, noisy galvanic vestibular stimulation, center of pressure, balance disorder, stochastic resonance

## Abstract

Balance disorders are a risk factor for falls in the elderly population. Balance control involving the complex interaction among nervous, muscular, and sensory systems should be maintained to keep an upright posture and prevent falls. Vestibular sensation is one of the main senses essential for postural control. Noisy galvanic vestibular stimulation (nGVS) is a noninvasive stimulation method for vestibular organs. Recently, it has received increasing attention for the treatment of balance disorders. However, the effect of balance disorders on stimulus effect during the implementation of nGVS remains unknown. Therefore, this study aimed to determine the effects of different floor surface and visual conditions on the stimulus effects of the nGVS intervention. In this study, two experiments were conducted with 24 participants (12 each for Experiments 1 and 2). In Experiment 1, nGVS (0.4 mA; 0.1–640 Hz) was performed in the open-eyes standing position on a solid surface (nGVS condition) and in the closed-eye standing position on a foam rubber (nGVS + foam rubber condition). In Experiment 2, sham stimulation was performed under the same conditions as in Experiment 1, except for nGVS. Center of pressure (COP) sway was measured in all participants with them standing with open eyes at Pre and Post-1 (immediately after the intervention) and Post-2 (10 min after the measurement of post-1). In Experiment 1, under the nGVS condition, COP sway was significantly reduced in Post-1 and Post-2 compared with Pre. However, no significant difference was observed among Pre, Post-1, and Post-2 under the nGVS + foam rubber condition. Furthermore, the intervention effect was significantly greater in the nGVS condition than in the nGVS + foam rubber condition. In contrast, in Experiment 2, the COP sway did not significantly differ among Pre, Post-1, and Post-2 under either condition. Based on the results of this study, nGVS was found to be effective with open-eyes standing on a solid surface.

## Introduction

Balance is an important predictor of falls among the elderly population. Falling has become very common and presents a substantial health problem among the elderly population owing to the overwhelming increase in human life expectancy (Wallmann, 2009). To maintain an upright posture and prevent falls, balance control involving the complex interaction among nervous, muscular, and sensory systems should be maintained. The visual, proprioceptive, and vestibular sensory systems provide feedback from the environment and contribute to balance control by facilitating interaction with the external world (Peterka, 2002; Gadkaree et al., 2016). Among these, the vestibular system senses angular and linear head accelerations in the space, which furnishes the central nervous system with information about self-motion, head position, and spatial orientation in relation to gravity (Cullen, 2012; Herssens and McCrum, 2019). However, vestibular system function declines with increasing age (Agrawal et al., 2012), and this can increase the risk of falls (Herdman et al., 2000; Agrawal et al., 2009).

Center of pressure (COP) measurement is one of the methods for assessing balance disorders (Pizzigalli et al., 2016). Previous studies reported higher values of path length and mean velocity of COP in the elderly than those in the younger population (Benjuya et al., 2004). In addition, this study has shown that fallers exhibit significant increases in COP sway path length and velocity compared with non-fallers (Melzer et al., 2004; Johansson et al., 2017). Noisy galvanic vestibular stimulation (nGVS) involves applying a weak noise electrical current to the vestibular end organs and their afferent nerves through electrodes placed bilaterally over the mastoid process (Fujimoto et al., 2019). Our cohort has shown that COP sway was improved in young and community-dwelling elderly participants who maintained an opened eye (on solid surface) while standing during nGVS (Inukai et al., 2018a,b). We also found that the effect of nGVS on decreasing COP sway persisted even after the end of the stimulation (Inukai et al., 2020). Conversely, Iwasaki et al. showed that the COP sway of individuals standing with closed eyes on foam rubber was reported to decrease during nGVS (Iwasaki et al., 2014). Additionally, the COP sway when standing with eyes closed on a foam rubber is considerably decreased by motor learning effect when repeatedly performed (Nordahl et al., 2000). Furthermore, the effect of balance training on a foam rubber has been reportedly greater than that of balance training on a solid surface (Hirase et al., 2015). Therefore, even in the nGVS, performing on a foam rubber may be more effective than performing on a solid surface. However, effects of floor surface (solid surface or foam rubber) and visual conditions (open or closed eyes) on the stimulatory effect of nGVS in decreasing COP sway are unclear. Although nGVS has been suggested to be a promising approach to improve balance function, effective stimulation protocols are not yet standardized (Herssens and McCrum, 2019). Therefore, this study aimed to clarify the effective stimulation condition of nGVS by performing nGVS in different conditions (floor surface and visual condition) and comparing the effects on COP sway after the intervention.

## Materials and Methods

### Participants

Two experiments were conducted in this study. Twelve participants (four men and eight women; mean age 20.75 ± 0.11 years) were recruited in Experiment 1 and another 12 participants (four men and eight women; mean age, 20.80 ± 0.13 years) in Experiment 2. Experiments 1 and 2 had different participants. None of them were taking medication or had a history of physical or neurological disorders. Participants were fully informed regarding the nature of the research and provided written informed consent before starting the experiment. This study was conducted in accordance with the Declaration of Helsinki and was approved by the ethics committee of Niigata University of Health and Welfare (17750–161007).

### Noisy Galvanic Vestibular Stimulation

Noisy galvanic vestibular stimulation was delivered using a DC-STIMULATOR PLUS (Eldith, NeuroConn GmbH, Ilmenau, Germany). A 1.5-cm-diameter circular electrode was used as a stimulating electrode and was applied to the mastoid process on both sides (Rt: anode, Lt: cathode). For nGVS, a random current level was generated for every sample (sampling rate, 1,280 samples/s). Random numbers were normally distributed, and the density function followed a bell-shaped curve (Terney et al., 2008; Moliadze et al., 2012). In Experiment 1, nGVS was performed at 0.4 mA (0.1–640 Hz), and in Experiment 2, sham stimulation (0 mA) was performed.

### Measurement of Center of Pressure

COP sway was measured for 30 s at 100 Hz in a standing position with legs together using a CFP400PA102RS (Leptrino, Nagano, Japan). Average COP sway path length, mediolateral (ML) mean velocity, and anteroposterior (AP) mean velocity were calculated (Inukai et al., 2018a,b, 2020).

## Experimental Procedures

### Experiment 1

**FIGURE 1:**
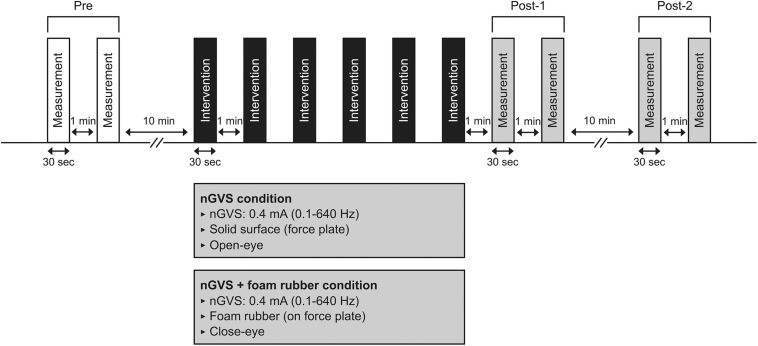
Experimental 1 procedure. Participants underwent COP measurements (30 s) at pre, post-1, and post-2. Interventions in either condition (30 s × 6 times) were performed between pre and post-1.

The procedure for Experiment 1 is shown in Figure 1. Participants underwent a total of six interventions after two COP sway measurements (open eyes) before the intervention (Pre). Two intervention conditions were used: nGVS and nGVS + foam rubber. In the nGVS condition, participants underwent 30-s nGVS in the open-eye standing position on a force plate. In the nGVS + foam rubber condition, participants underwent nGVS in the closed-eye standing position on a foam rubber placed on a force plate. In each condition, two COP sway measurements were performed immediately after the six interventions (Post-1). In addition, two COP sway measurements were performed 10 min after the Post-1 measurement (Post-2). Half of the participants performed the nGVS condition first, and the other half performed the nGVS + foam rubber condition first. The condition to be performed first was randomly selected in half, and the interval between measurements was at least 7 days. From the measurement of Pre to the start of Intervention, a 10-min rest period was provided between Post-1 and Post-2, with a 1-min rest period between interventions. All subjects were interviewed after the experiment and asked if they felt the stimulation.

### Experiment 2

**FIGURE 2:**
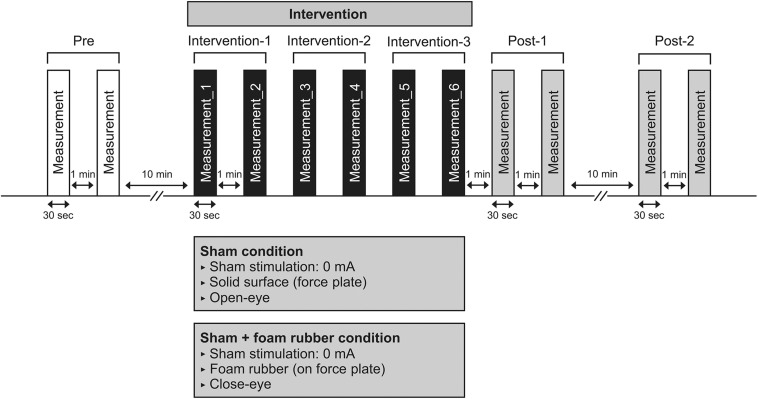
Experimental 2 procedure. Participants underwent COP sway measurements during the intervention six times after COP sway measurements in pre. COP sway measurements at post-1 were performed immediately after the intervention, and a further COP sway measurement (post-2) was performed 10 min after.

The procedure for Experiment 2 is detailed in Figure 2. As in Experiment 1, six interventions were performed after COP sway measurements of Pre were performed. The two intervention conditions were the same as in Experiment 1; however, sham stimulation (0 mA) was used. In Experiment 2, we performed COP sway measurements during each intervention and calculated at Interventions 1, 2, and 3. After the intervention, COP sway measurements of Post-1 and Post-2 were performed using the same procedure as in Experiment 1. The condition to be performed first was randomly selected in half, and the interval between measurements was at least 7 days.

### Statistical Analysis

The IBM SPSS, Version 21 (IBM Corp., Armonk, NY, United States) was used for all statistical analyses, with the significance level set at 5%.

### Experiment 1

Sway path length, ML mean velocity, and AP mean velocity at pre, post-1, and post-2 were compared using two-way repeated measures analysis of variance (ANOVA) as follows: [time (pre, post-1, post-2)] × [intervention (nGVS, nGVS + foam rubber)]. A post-hoc analysis using Bonferroni’s methods was used if a significant difference in the interaction was observed. Further, the intervention effect for each condition was calculated using the pre value and post-value: (Δ intervention effect-1 = [pre] − [post-1]), (Δ intervention effect-2 = [pre] − [post-2]). In addition, intervention effects 1 and 2 were compared between the two conditions using a paired *t*-test.

### Experiment 2

Sway path length, ML mean velocity, and AP mean velocity at pre, interention-1, intervention-2, intervention-3, post-1, and post-2 were compared using two-way repeated measures ANOVA as follows: [time (pre, intervention-1, intervention-2, intervention-3, post-1, post-2)] × [intervention (sham, sham + foam rubber)]. A post-hoc analysis using Bonferroni’s methods was used if a significant difference in the interaction was observed.

## Results

### Experiment 1

**FIGURE 3:**
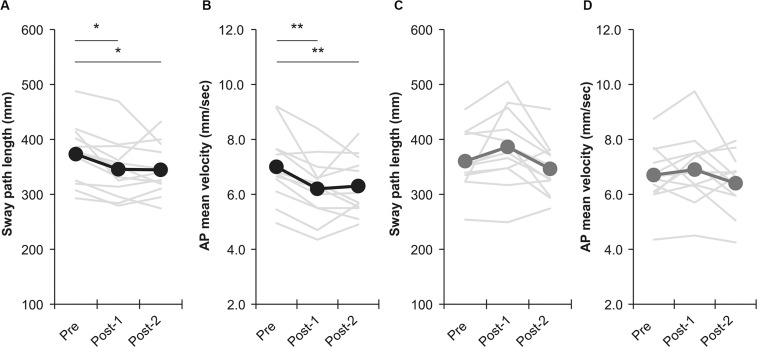
Effects of intervention in the nGVS condition and nGVS + foam rubber condition. **(A)** Sway path length of nGVS condition. **(B)** AP mean velocity of nGVS condition. **(C)** Sway path length of nGVS + foam rubber condition. **(D)** AP mean velocity of nGVS + foam rubber condition. Error bars indicate SE. *p < 0.05, **p < 0.01.

**FIGURE 4:**
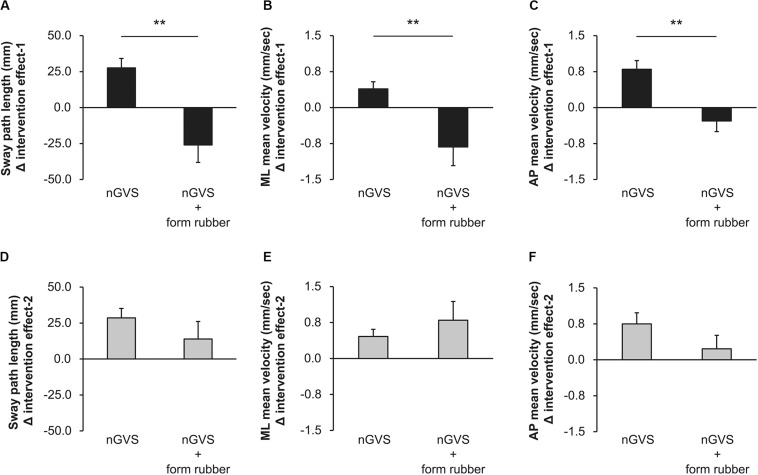
Values of the intervention effect for each condition. **(A)** Intervention effect-1 of sway path length. **(B)** Intervention effect-1 of ML mean velocity. **(C)** Intervention effect-1 of AP mean velocity. **(D)** Intervention effect-2 of sway path length. **(E)** Intervention effect-2 of ML mean velocity. **(F)** Intervention effect-2 of AP mean velocity. Error bars indicate SE. **p < 0.01.

Among all participants, only one experienced the noise current stimulation, whereas other participants did not experience the noise current stimulation during stimulation. Table 1 shows the results of two-way repeated measures ANOVA. Sway path length and AP mean velocity showed a significant main effect (time) and interaction (intervention × time). Table 2 shows the value of COP sway measurement and intervention effect values for each intervention condition. In the nGVS condition, the sway path length and AP mean velocity significantly decreased in post-1 and post-2 compared with pre (Figures 3A,B). In the nGVS + foam rubber condition, the sway path length and AP mean velocity was not significantly different in post-1 and post-2 compared with pre (Figures 3C,D). The mean value of the intervention effect-1 ± standard error was nGVS condition; the sway path length was 27.6 ± 6.6 mm, ML mean velocity was 0.4 ± 0.2 mm/s, AP mean velocity was 0.8 ± 0.2 mm/s, nGVS + foam rubber condition; the sway path length was −26.0 ± 12.1 mm, the ML mean velocity was −0.8 ± 0.4 mm/s, and the AP mean velocity was −0.3 ± 0.2 mm/s. The mean value of the intervention effect-2 ± standard error was nGVS condition; sway path length was 28.6 ± 11.0 mm, ML mean velocity was 0.5 ± 0.3 mm/s, AP mean velocity was 0.8 ± 0.2 mm/s, nGVS + foam rubber condition; the sway path length was -26.0 ± 12.1 mm, ML mean velocity was −0.8 ± 0.4 mm/s, and AP mean velocity was 0.2 ± 0.3 mm/s. The intervention effect-1 was significantly higher in the nGVS condition than in the nGVS + foam rubber condition for all parameters (Figures 4A,C). The intervention effect-2 showed no significant difference between two conditions for all parameters (Figures 4D,F).

**TABLE 1:** The results of two-way repeated measures ANOVA for Experiments 1 and 2.

	**Intervention**		**Time**		**Intervention × Time**	
	***F*-value**	***P*-value**	***F*-value**	***P*-value**	***F*-value**	***P*-value**
**Experiment 1**						
Sway path length	0.724_(1, 11)_	0.413	4.019_(2, 22)_	0.033	4.505_(1.192, 13.110)_	0.023
ML mean velocity	1.360_(1,11)_	0.268	1.382_(2, 22)_	0.129	3.722_(2.194, 14.827)_	0.063
AP mean velocity	0.640_(1, 11)_	0.441	4.148_(2, 22)_	0.030	4.806_(2, 22)_	0.019
**Experiment 2**						
Sway path length	100.992_(1, 11)_	0.000	80.605_(1.634, 17.972)_	0.000	86.779_(1.571, 17.278)_	0.000
ML mean velocity	93.078_(1,11)_	0.000	86.199_(1.443, 15.869)_	0.000	81.381_(1.385, 15.234)_	0.000
AP mean velocity	109.193_(1, 11)_	0.000	78.302_(1.850, 20.349)_	0.000	82.316_(1.898, 20.873)_	0.000

*AP = anteroposterior; ML = mediolateral; ANOVA = analysis of variance.*

**TABLE 2:** The COP sway values and intervention effects in Experiment 1.

	**Value of COP sway measurement**			**Intervention effect values**	
	**Pre**	**Post-1**	**Post-2**	**Intervention effect-1**	**Intervention effect-2**
**nGVS**					
Sway path length (mm)	373.1 ± 16.0	345.5 ± 15.5	344.5 ± 13.5	27.6 ± 6.6	28.6 ± 11.0
ML mean velocity (mm/s)	7.7 ± 0.4	7.3 ± 0.4	7.3 ± 0.3	0.4 ± 0.2	0.5 ± 0.3
AP mean velocity (mm/s)	7.1 ± 0.4	6.3 ± 0.3	6.3 ± 0.3	0.8 ± 0.2	0.8 ± 0.2
**nGVS + foam rubber**					
Sway path length (mm)	360.0 ± 15.6	386.3 ± 20.2	346.4 ± 14.3	−26.0 ± 12.1	14.0 ± 18.1
ML mean velocity (mm/s)	7.5 ± 0.4	8.3 ± 0.6	7.4 ± 0.4	−0.8 ± 0.4	0.8 ± 0.4
AP mean velocity (mm/s)	6.7 ± 0.3	7.0 ± 0.4	6.5 ± 0.3	−0.3 ± 0.2	0.2 ± 0.3

*mean ± standard error; AP = anteroposterior; ML = mediolateral.*

### Experiment 2

**FIGURE 5:**
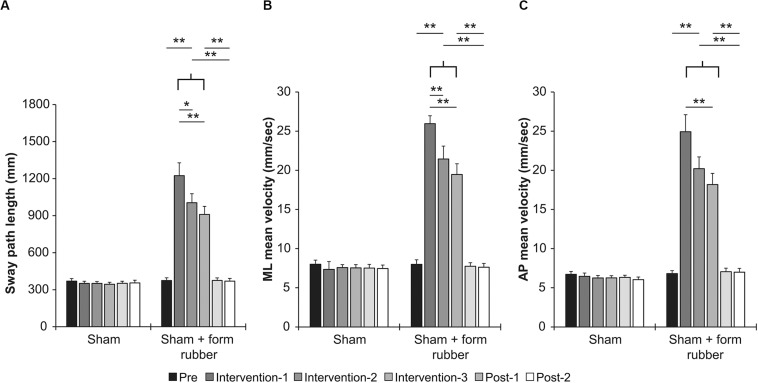
Effects of intervention in the sham condition and sham + foam rubber condition. **(A)** Sway path length. **(B)** ML mean velocity. **(C)** AP mean velocity. Error bars indicate SE.*p < 0.05, **p < 0.01.

Table 1 shows the results of two-way repeated measures ANOVA. Significant main effects (intervention and time) and interactions (intervention × time) were found for all parameters. Table 3 shows the value of COP sway measurement for each intervention condition. In the sham condition, no significant changes were observed in all parameters (Figures 5A,C). In the sham + foam rubber condition, all parameters were significantly increased during the intervention (interventions 1, 2, and 3) compared with pre. Moreover, intervention 1 was significantly higher than interventions 2 and 3, but without significant difference between interventions 2 and 3. Moreover, post-1 and post-2 were significantly lower than during the interventions (interventions 1, 2, and 3), but without significant difference between them and pre (Figures 5A,C).

**TABLE 3:** The COP sway values in Experiment 2.

	**Pre**	**Intervention-1**	**Intervention-2**	**Intervention-3**	**Post-1**	**Post-2**
**Sham**						
Sway path length (mm)	369.4 ± 21.0	351.3 ± 17.3	351.1 ± 15.5	344.4 ± 15.9	351.7 ± 17.0	355.5 ± 22.0
ML mean velocity (mm/s)	8.0 ± 0.5	7.4 ± 0.4	7.6 ± 0.4	7.5 ± 0.4	7.5 ± 0.5	7.5 ± 0.5
AP mean velocity (mm/s)	6.7 ± 0.4	6.5 ± 0.4	6.3 ± 0.3	6.3 ± 0.3	6.3 ± 0.3	6.0 ± 0.3
**Sham + foam rubber**						
Sway path length (mm)	374.2 ± 22.4	1223.7 ± 103.9	1004.4 ± 73.5	909.7 ± 65.7	375.2 ± 21.0	369.5 ± 22.6
ML mean velocity (mm/s)	8.0 ± 0.6	26.0 ± 2.3	21.4 ± 1.7	19.5 ± 1.4	7.7 ± 0.5	7.6 ± 0.5
AP mean velocity (mm/s)	6.8 ± 0.4	24.9 ± 2.2	20.2 ± 1.5	18.2 ± 1.4	7.1 ± 0.4	7.0 ± 0.5

*mean ± standard error; AP = anteroposterior; ML = mediolateral.*

## Discussion

In this study, it was clarified that the COP sway decreased when nGVS was performed in open-eyes standing position on a solid surface. In Experiment 2, the COP sway did not decrease during repeated standing with sham stimulation, suggesting that this was not a motor learning effect but a stimulation effect of nGVS. In contrast, after repeated nGVS or sham stimulation with eyes closed on a foam rubber, the COP sway of post remained unchanged. Furthermore, the intervention effects of Experiment 1 were greater in the nGVS condition (open eyes/solid surface) than that in the nGVS + foam rubber condition. These results suggest that nGVS is more effective in open-eyes standing on a solid surface than that in closed-eyes standing on a foam rubber.

Usually, the presence of noise in a sensory system is believed to have detrimental effects on the system’s ability to detect signals and to process the incoming flow of information. However, evidence that an appropriate amount of noise can improve both the detection and transmission of weak input signals in nonlinear systems is increasing (Moss et al., 2004; McDonnell and Ward, 2011; Wuehr et al., 2017). Stochastic resonance, which is considered to be related to nGVS’ role in decreasing the COP sway, is the mechanism underlying this phenomenon. During the application of nGVS, the neuronal activity of the primary vestibular afferents (semicircular canals and otolith organs) and vestibular hair cells is thought to be altered via stochastic resonance, i.e., noise (Gensberger et al., 2016; Herssens and McCrum, 2019). Moreover, nGVS effectively lowers the vestibular threshold to elicit balance-related reflexes (vestibulospinal reflex) that are required to adequately regulate postural equilibrium (Schniepp et al., 2018; Wuehr et al., 2018). This study suggests that stochastic resonance generated by the noisy current stimulation improves vestibular function and decreases COP sway.

A previous study reported that COP sway when standing with eyes close on foam rubber considerably decreased by the learning effect when repeatedly performed (Nordahl et al., 2000). In this study, a significant decrease in COP sway was also observed in Experiment 2 with repeated closed-eyes standing position on a foam rubber (Interventions 1–3). However, no significant difference was observed in Post-1 and Post-2 COP sway measured with the open-eyes standing position on a solid surface compared with Pre. These results show that the learning effect of repeated closed-eyes standing position on a foam rubber is suggesting the absence of spillover effect on the reduction of COP sway during open-eye standing position on the solid surface. Furthermore, in Experiment 1, nGVS with closed-eyes standing position on foam rubber did not decrease the COP sway with open-eyes standing position on a solid surface after the intervention. A previous study reported that COP sway when standing with eyes closed on a foam rubber decreased when nGVS was performed when standing with eyes closed on a foam rubber (Iwasaki et al., 2014). In this study, unlike the previous study, COP sway measurements were performed after the nGVS, not during nGVS. Additionally, in the previous study, the COP sway was measured while standing with the eyes closed on a foam rubber, whereas, in this study, the COP sway was measured while standing with eyes open on a solid surface. The fact that the COP sway measurement timing and environment were different from those of the previous study may influence the difference the results. Furthermore, in a previous study, the optimal stimulus intensity of nGVS for decreasing COP sway with closed-eyes standing position on a foam rubber was 0.28 ± 0.03 mA, and the stimulation effect of nGVS above the optimal stimulus intensity reduced (Iwasaki et al., 2014). In this study, the stimulation intensity of nGVS was slightly higher than that in a previous study; therefore, it is possible that the COP sway after nGVS did not decrease with the eyes closed on a foam rubber.

This study has several limitations. First, nGVS was performed only with one stimulus intensity of 0.4 mA. We have reported that 0.4 mA nGVS is suitable for reducing COP sway in healthy young people and community-dwelling elderly people (Inukai et al., 2018a,b, 2020). In this study, the COP sway post status did not decrease even if 0.4 mA nGVS was performed with closed-eyes standing position on a foam rubber. However, it is possible that the results may be different by changing the stimulation intensity of nGVS. In the future, the effect of nGVS should be verified at different stimulus intensities. Second, all participants were healthy young individuals. In previous studies, we have demonstrated that the effects of nGVS are similar in young healthy people and community-dwelling elderly people (Inukai et al., 2018a,b). In the future, verification should be performed in different participants, but similar results may be obtained even for the community-dwelling elderly. In addition, results of this study verified only the immediate effect after a 1-day intervention. Considering that balance training on a foam rubber for several days is effective (Hirase et al., 2015), it is possible that nGVS on a foam rubber for several days may have a significant stimulatory effect. Therefore, it is necessary to verify the intervention effect over multiple days in the future. Furthermore, this study showed that the COP sway at post-intervention was decreased when nGVS was performed under open-eyes standing on solid surface conditions. However, since the intervention and measurement conditions were limited, the most effective intervention for nGVS was unclear. In the future, it is necessary to conduct a complete factorial approach that combines all floor surfaces (solid surface or foam rubber) and visual conditions (open or closed eyes) and to verify the effect of the most effective intervention for nGVS.

## Conclusion

Based on the results of this study, the stimulation effect of nGVS (0.4 mA) was found to decrease the COP sway depending on the floor condition and visual state. This study also suggested that nGVS with open eyes on a solid surface is effective in decreasing COP sway.

## Data Availability Statement

All datasets presented in this study are included in the article/supplementary material.

## Ethics Statement

The study involving human participants was conducted in accordance with the Declaration of Helsinki and was approved by the ethics committee of Niigata University of Health and Welfare (17750–161007). The patients/participants provided their written informed consent to participate in this study.

## Author and Contributions

HO conceived the study and designed the experiment. YI and MS conducted the experiments. SM and NO interpreted the data. YI and MS performed the statistical analysis. SM and NO helped writing and revising the manuscript. YI and HO wrote the manuscript. All authors read and approved the final manuscript.

## Conflict of Interest

The authors declare that the research was conducted in the absence of any commercial or financial relationships that could be construed as a potential conflict of interest.
